# The Stack Optimization of Magnetic Heterojunction Structures for Next-Generation Spintronic Logic Applications

**DOI:** 10.3390/ma16196418

**Published:** 2023-09-26

**Authors:** Jaehun Cho, Jinyong Jung, Seong Bok Kim, Woo Ri Ju, Da Hyeon Kim, Myunghwan Byun, June-Seo Kim

**Affiliations:** 1 Division of Nanotechnology, Daegu Gyeongbuk Institute of Science and Technology (DGIST), Daegu 42988, Republic of Korea; 2 Department of Physics and Chemistry, Daegu Gyeongbuk Institute of Science and Technology (DGIST), Daegu 42988, Republic of Korea; 3 Department of Materials Engineering, Keimyung University, Daegu 42601, Republic of Korea

**Keywords:** spin torque majority gate, Dzyaloshinskii–Moriya interaction, micromagnetic simulations, perpendicular magnetic anisotropy, interlayer exchange coupling, Ruderman–Kittel–Kasuya–Yosida interaction

## Abstract

Magnetic heterojunction structures with a suppressed interfacial Dzyaloshinskii–Moriya interaction and a sustainable long-range interlayer exchange coupling are achieved with an ultrathin platinum insertion layer. The systematic inelastic light scattering spectroscopy measurements indicate that the insertion layer restores the symmetry of the system and, then, the interfacial Dzyaloshinskii–Moriya interaction, which can prevent the identical magnetic domain wall motions, is obviously minimized. Nevertheless, the strong interlayer exchange coupling of the system is maintained. Consequently, synthetic ferromagnetic and antiferromagnetic exchange couplings as a function of the ruthenium layer thickness are observed as well. Therefore, these optimized magnetic multilayer stacks can avoid crucial issues, such as domain wall tilting and position problems, for next-generation spintronic logic applications. Moreover, the synthetic antiferromagnetic coupling can open a new path to develop a radically different NOT gate via current-induced magnetic domain wall motions and inversions.

## 1. Introduction

At the interface between ferromagnets and heavy metals in heterojunction structures, strong spin–orbit coupling (SOC) gives rise to various intriguing phenomena, such as surface anisotropy, the Dzyaloshinskii–Moriya interaction (DMI), and proximity-induced magnetic moments [1,2,3]. These magnetic properties, which exist at the well-defined interfaces created by state-of-the-art nanoscale thin-film growth technologies, offer numerous applications [4,5]. For instance, they can be utilized in the development of next-generation non-volatile memory devices based on chiral magnetic domain walls (DWs) or magnetic skyrmions [6,7,8,9]. In recent times, there has been significant research focus on the development of logic circuits or logic devices which operate at ultra-high speeds and ultra-low power consumption, utilizing chiral magnetic DWs and skyrmions [10,11,12,13,14]. These novel magnetic structures have attracted great interest due to their potential to revolutionize information processing technologies.

However, it is extremely challenging to achieve an ultrathin magnetic heterojunction structure that simultaneously satisfies diverse requirements for various magnetic properties due to the conflicting nature of the device characteristics. For instance, interlayer exchange coupling (IEC) enables the easiest creation of domain wall reversal devices. However, the occurrence of DMI during the formation of magnetic heterojunction structures compromises the reliability of DW reversal devices. Therefore, the systematic study of the strong correlation between IEC and DMI with various heterojunction structures is crucial for the development of chiral DW motion-based logic applications.

**Figure 1:**
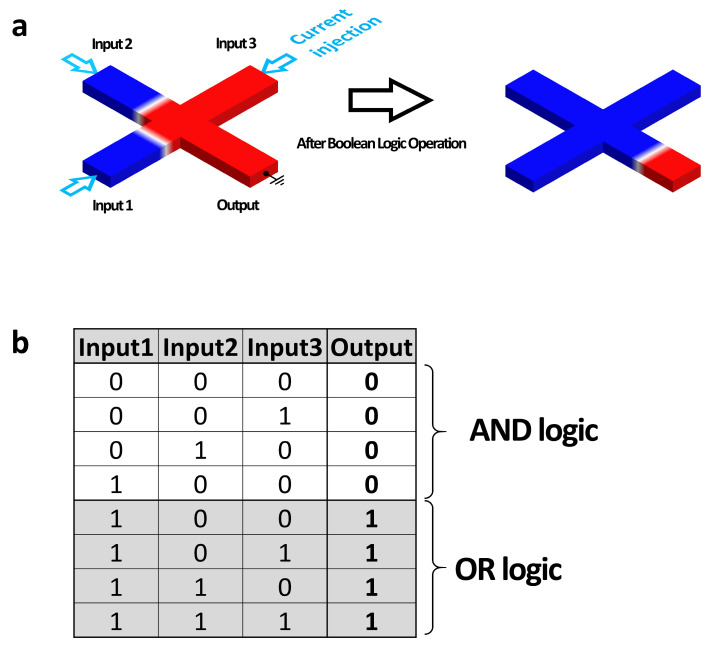
(**a**) Schematic of the spin configuration and the operation mechanism of the STMG device. There are three input and one output terminals. The arrows show the directions of the electrical current. Red and blue magnetic domains indicate the perpendicularly magnetized nanowires, with the magnetizations pointing up and down, respectively. The magnetic DWs are located between two magnetic domains. (**b**) The truth table of the STMG.

Here, we study the optimal conditions of the magnetic multilayer structures for the next-generation logic devices, which are so-called “spin torque majority gates [12,15,16] (STMGs)” based on current-induced magnetic DW motions. Figure 1a presents a schematic figure of the STMG device. The STMG device consists of three individual components: the DW input, that generates DWs by a strong SOC from the heavy metal; the majority gate, that is responsible for logic operations based on DW motions; and the DW inverter, which is one of the key components for realizing a NOT gate. Realizing a NOT gate based on the magnetic DW motion poses significant challenges, as the magnetizations of the DWs need to be reversed when passing through the inverter. To address this issue, we employ either the Ruderman–Kittel–Kasuya–Yosida (RKKY) interaction or interlayer exchange coupling (IEC) to successfully implement a DW inverter in our study.

The majority gate is a logic gate that outputs the value of the majority among three individual inputs. For the case of the STMG, the three inputs are the chiral magnetic DWs. The crucial factor of the STMG device is that all three input values participate in the operation with equal probability and opportunity. This requirement emphasizes the necessity for equal weighting and consideration of each input, allowing for fair and unbiased decision making within the majority gate. Figure 1b shows the majority gate truth table. When the DW input at each input represent “1”, the DW does not output represents “0”. The main advantage of the majority gate is that a single majority gate can realize AND gate and OR gate due to three individual inputs.

For optimizing the magnetic heterostructure for the next-generation STMG devices, several key issues should be considered: (i) the successful DW nucleation via the field-free SOT switching mechanism; the system needs to have a certain DMI energy density at the DW input part. (ii) The magnetic DWs should be displaced and stopped at identical positions for the STMG operation. On the other hand, it is well known that the interfacial DMI energy occurs the tilting angle of the DW, which can prevent to stop the DWs at the cross area of the STMG device depicted in Figure 1a. Therefore, a reduction of the DMI energy is necessary for the STMG operation. (iii) For the DW inverter, a strong IEC is required and the upper and lower magnetic layers should have similar magnetic properties, such as surface anisotropy energy density and saturation magnetization, since the input and output DWs after the NOT gate operation should be identical.

Here, we successfully modulate the DMI energy densities by inserting an ultrathin Pt layer between Co and Ru layers in a Ta/Pt/Co/Ru/Pt/Co/Pt structure. Both the upper and lower magnetic layers have a strong perpendicular magnetic anisotropy (PMA) energy and the interfacial DMI energy is sufficiently reduced due to the Pt layer insertion. Moreover, periodic synthetic ferromagnetic and antiferromagnetic coupling behaviors as a function of the Ru layer thickness via a strong IEC are observed as well.

## 2. Materials and Methods

The magnetic multilayers Ta (5.0)/Pt (4.0)/Co (*t*_Co_)/Pt (*t*_Pt_ = 0.0, 0.2, and 0.4)/Ru (3.0) (thickness in nm) were deposited by employing a wedge-type DC magnetron sputtering system, and the working conditions were carefully controlled for all thin films, with an argon pressure of 2 mTorr. The width and length of the substrates were 10 mm and 20 mm, respectively. As the linear shadow mask was moved along the length direction of the substrate with a certain velocity, cobalt layers grew to form a wedge-type layer. The thickness of the cobalt layer (*t*_Co_) was varied from 0.5 nm to 2.5 nm, and the thicknesses of the Pt insertion layers (*t*_Pt_) were selected as 0 nm, 0.2 nm, and 0.4 nm to reduce the iDMI energy densities. All samples were grown on 100 nm SiO_2_ substrates.

Micromagnetic simulations using “Mumax3” were performed to investigate the magnetic DW motions. Two perpendicularly magnetized nanowires with dimensions of 5000 nm (length) × 100 nm (width) × 0.6 nm (thickness) were chosen, and they overlapped at the center of the cross. The cell size was fixed at 1 nm × 1 nm × 0.6 nm. For the micromagnetic simulations, the material parameters used were a saturation magnetization (*M*_s_) of 1090 kA/m, an exchange stiffness constant of 10 pJ/m, a gyromagnetic ratio of 1.90 × 10^11^ Hz/T, a PMA energy density of 1.25 MJ/m^3^, and a Gilbert damping constant of 0.5. The values of the simulation parameters were taken from Reference [17].

Polar magneto-optical Kerr effect (MOKE) spectroscopy was used to measure the magnetic hysteresis as a function of the external magnetic fields. A SQUID-VSM was also employed to measure the saturation magnetization, which can be directly compared with the results from the BLS measurements. To determine the iDMI energy density, an inelastic light scattering technique, known as Brillouin light scattering (BLS) measurements, was systematically performed with a multi-pass tandem Fabry–Perot (FP) interferometer [18]. The wavelength and power of the *p*-polarized single longitudinal mode laser were 532 nm and 170 mW, respectively. The back-scattered photons were collected via a camera lens and passed through the FP interferometer.

## 3. Results and Discussion

### 3.1. Micromagnetic Simulations

**Figure 2:**
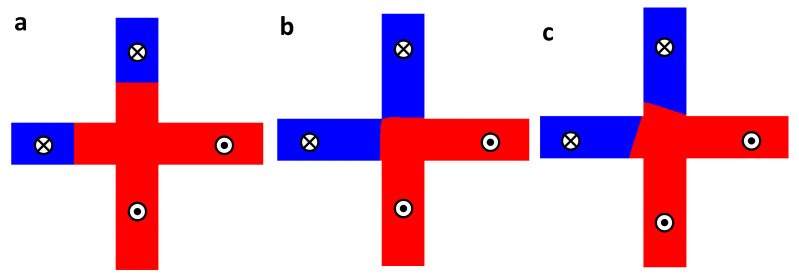
(**a**) Two individual DWs are nucleated 150 nm away from the center of the junction area. (**b**,**c**) The field-induced magnetic DW motions without (**b**) and with (**c**) the iDMI contribution.

It is noted that all magnetic DWs should meet at the edges of the cross-shape junction area, which is the main assumption of the STMG device for the equal opportunities of the logic operation. Figure 2 shows the micromagnetic simulation results of the STMG device with or without the contribution of the iDMI energy density, performed using Mumax3 [19]. As an initial spin configuration, two individual Néel type DWs are nucleated at a certain distance (150 nm) from the center of the junction, as shown in Figure 2a. In order to DW tilting behavior at the border of the junction caused by iDMI, we choose values of the iDMI of 0 and 2 mJ/m^2^, depicted in Figure 2b,c, respectively. To simplify observation of the spin-torque effect, spin-transfer torque-driven DW motions are performed. DWs start to move along the nanowire when a current density of 5 × 10^11^ A/m along the *z*-direction is applied to the system. Consequently, DWs approach the center of the junction area without a tilting angle due to a negligible iDMI energy density, and two DWs can meet at the edges of the junction. This state is read to the equivalent logic operation. On the other hand, for the case of the system with *D* = 2 mJ/m^2^, DWs are moved to the center with a certain DW tilting angle, which depends on the strength of the iDMI energy density. In this case, DWs do not reach the border of the junction area due to a chirality-induced magnetic DW tilting. Therefore, a reduction in the iDMI energy density should be needed for the STMG operation.

### 3.2. Magnetic Properties

**Figure 3:**
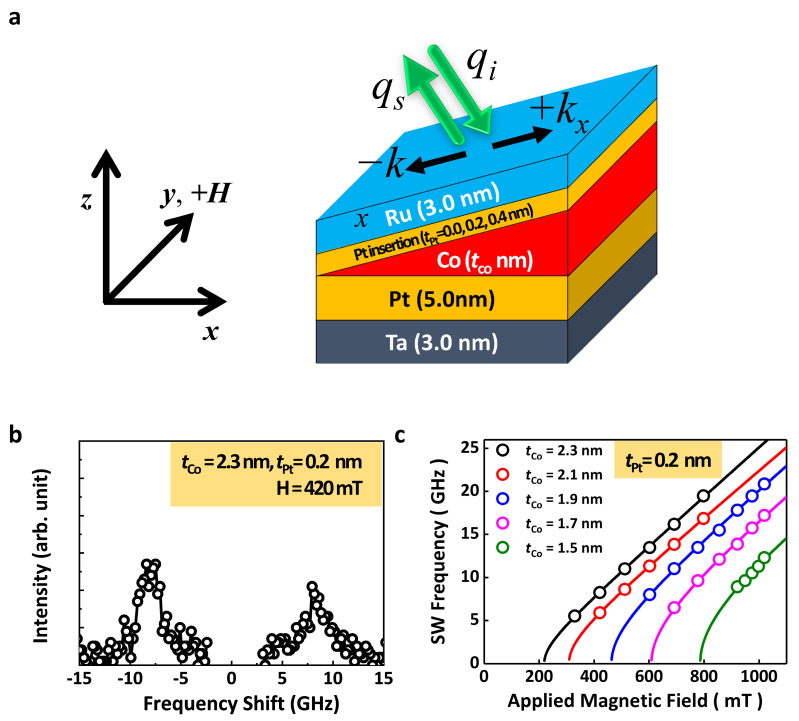
(**a**) Back-scattering geometry for Brillouin scattering measurement with coordinates and axes. Also, schematic picture of the Co wedge sample structure with an inserted Pt layer. (**b**) The BLS spectrum with a magnetic field = 420 mT along the *y*-direction. (**c**) The applied magnetic field dependence of the spin wave frequencies for various Co thicknesses with a 0.2 nm thick Pt insertion layer. The open symbols are experimental results, and the solid lines represent the least squares fitting curve using Equation (1).

Figure 3a shows a schematic picture of the Co wedge sample structure with an inserted Pt layer to investigate the magnetic properties. Back-scattering geometry was used to observe the spinwave (SW) frequency, as shown in Figure 3a. We used an applied magnetic field along the *y*-direction with a fixed *k_x_* = 0.0167 nm^−1^. When the magnetizations of the systems are saturated to the film surface and perpendicular to the scattering plane with the wavevector of incident lights, a surface mode (the so-called “Damon–Eshbach (DE) mode”) of SWs is propagated in this geometry. A BLS is a versatile piece of equipment to determine various magnetic properties by fitting the measured SW resonance frequencies as a function of the applied magnetic field via Kittel’s equation. The typical spectrum of a Ta/Pt/Co (2.3 nm)/Pt (0.2 nm)/Ru sample is shown Figure 3b, with an applied magnetic field of 420 mT along the *y*-direction. Figure 3c indicates the propagating SW frequencies when increasing the applied fields as a function of *t*_Co_, with *t*_Pt_ fixed at 0.2 nm. Since there are frequency differences (Δ*f*) between Stokes and anti-Stokes SW frequencies due to the iDMI energy density, the averaged SW frequencies are used. The SW frequencies (*f*_SW_) as a function of the magnetic fields for various cobalt thicknesses (*t*_Co_) are the following [20]:

(1) $$f_{SW}=\frac{\gamma }{2\pi }\sqrt{\left(H\left(H+\frac{2K_{eff}}{{\mu_{0}\cdot M}_{S}}\right)\right)},$$

where *γ* is the gyromagnetic ratio (=1.90 × 10^11^ Hz/T), *H* is the applied magnetic field, and *K*_eff_ is the effective PMA. In a DE mode of SW frequency in a Co thin film, however, the contribution of exchange energy is below 0.2 GHz. This result is below the uncertainty of our experimental system (~0.3 GHz). Therefore, the exchange stiffness energy is negligible in this study. In Figure 3c, all opened circles and lines are the measured data and lines are fit using Equation (1). It is seen that the SW frequencies increase with increasing *t*_Co_ and the fitting curves and the experimental data are in good agreement with each other.

**Figure 4:**
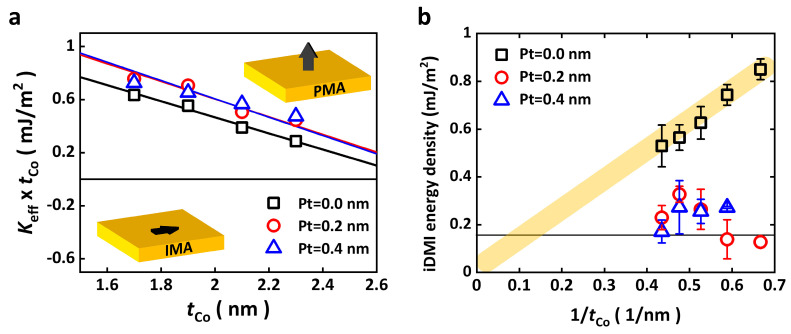
(**a**) $K_{eff}\times t_{Co}$ vs. $t_{Co}$ plot for the Pt inserted layer with a linear fitting line using Equation (2). The black open rectangles, red open circles, and blue open triangles represent *t*_Pt_ = 0, 0.2, and 0.4 nm, respectively. The positive $K_{eff}\times t_{Co}$ region means the easy axis is perpendicular to the plane. (**b**) The iDMI energy density as a function of $t_{Co}^{-1}$ for the Pt insertion layer with error. The black open rectangles, red open circles, and blue open triangles represent *t*_Pt_ = 0, 0.2, and 0.4 nm, respectively. The black solid line represents the experimental limit (=0.16 mJ/m^2^).

The first main purpose of this study is to suppress the iDMI energy density while enhancing the PMA energy (or surface anisotropy energy) by introducing Pt insertion layers. To determine the surface anisotropy energy density (*K*_s_), $K_{eff}\times t_{Co}$ versus $t_{Co}$ for each structure is plotted with linear fit lines in Figure 4a. The correlation between $K_{eff}\times t_{Co}$ and $K_{s}$ is expressed as:

(2) $$K_{eff}\times t_{Co}=2K_{s}-\left(\frac{1}{2}\mu_{0}M_{S}^{2}\right)\cdot t_{Co},$$

where *K*_s_ is the averaged surface magnetic anisotropy energy density of the top and bottom interfaces. To determine a precise value of *M_s_*, Ta (5.0 nm)/Pt (4.0 nm)/Co (1.7, 1.9, 2.1, and 2.3 nm)/Pt (*t*_Pt_ = 0.0, 0.2, and 0.4 nm)/Ru (3.0 nm) structured thin films are fabricated and a superconducting quantum interference device vibration sample magnetometer (SQUID-VSM) is used. The *K_s_* can be determined from the *y*-intercept of the linear fitting lines using the obtained *M_s_* values. The *K*_s_ values are increased by the insertion of a Pt layer, from 0.84 ± 0.01 (*t*_Pt_ = 0.0 nm) to 0.99 ± 0.03 mJ/m^2^ (*t*_Pt_ = 0.2 nm). The *M_s_* values for the whole sample are slightly increased by the insertion of a Pt layer, from 982 ± 47 (*t*_Pt_ = 0.0 nm) to 1049 ± 33 kA/m (*t*_Pt_ = 0.2 nm). The increase in *M_s_* values can be explained as the proximity-induced magnetic moment due to a strong spin–orbit coupling at the Co/inserted Pt interface. The determined *M_s_* and *K_s_* values are summarized in Table 1.

We now discuss the iDMI. The non-reciprocal SW dispersion relations is a consequence of the iDMI. The iDMI energy density is given by the measured SW frequency difference (Δ*f*) between SW creation (Stokes) and annihilation (anti-Stokes) processes, as shown below [21,22].

(3) $$\left|\Delta f\right|=\frac{2\gamma \left|D\right|}{\pi M_{s}}k_{x}$$

Here, $\left|D\right|$ is the absolute iDMI energy density, and *k_x_* the wavevector of SW. We used the *M_s_* value determined from the SQUID-VSM results. From the external magnetic field dependence measurements, we investigated the iDMI energy density as a function of $t_{Co}^{-1}$, as shown in Figure 4b with error bars. The result from the sample with no Pt insertion (*t*_Pt_ = 0.0 nm) shows inverse proportionality, which is similar to our previous results [20,21,22]. However, the values of iDMI of the Pt insertion samples are much smaller than for the no Pt insertion sample. This value decreased under technical limit of $\left|D\right|$ (~0.16 mJ/m^2^) measured by BLS [21] at *t*_Co_ = 1.5 nm and 1.7 nm with the 0.2 nm Pt insertion samples. In Table 1, the averaged *D*_s_ (=*D* × *t*_Co_) is also listed with standard deviations. The value of *D*_s_ is similar to in our previous works [20,21,22,23,24]. However, the *D*_s_ value of the Pt insertion samples are significantly suppressed. We propose a possible scenario that the $\left|D\right|$ with the Pt insertion layer is reduced by the presence of Pt atoms at the top and bottom layers. It is well known that the iDMI is caused by the effect of structural inversion asymmetry. There is a chance that the existence of the iDMI from the different interfaces and thicknesses of the Pt/Co and Co/Pt insertion layers [25,26]; however, a reduction in the iDMI is expected due to its symmetric structure, when Co and Pt at the Pt insertion layer undergo intermixing in the case of ultrathin Pt insertion. Without detailed interface analysis, it is hard to conclude the physical origin of the decrease in the $\left|D\right|$ for the Pt insertion layer samples.

### 3.3. Interlayer Exchange Coupling

**Figure 5:**
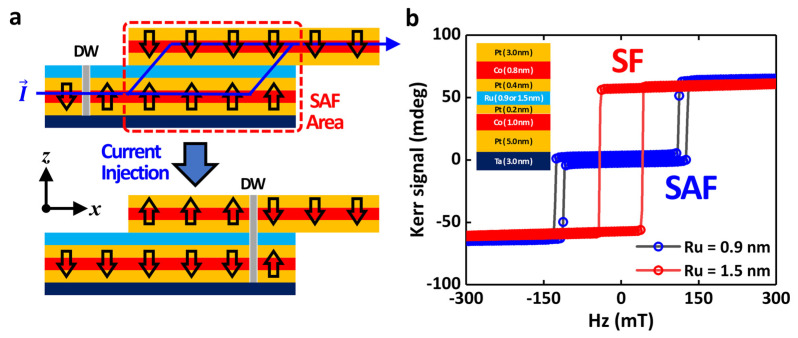
(**a**) Schematic of the IEC-based magnetic DW inverter. The arrows show the directions of the magnetizations. (**b**) Polar MOKE spectroscopy results. Blue and red circles indicate the synthetic antiferromagnetic (SAF) coupling (Ru = 0.9 nm) and the synthetic ferromagnetic (SF) coupling (Ru = 1.5 nm), respectively.

A radically different approach to fabricating a DW inverter is schematically depicted in Figure 5a. Since the system with a suppressed iDMI energy density is hard to make a DW inverter using the chiral coupling between adjacent magnetic domains, an SAF structure is considered in this study. As shown, two magnetic multilayers are vertically located and connected by the insertion layer in a certain area. In the overlapping area (SAF area), two perpendicular magnetizations are antiferromagnetically coupled with each other due to a strong IEC. The current-induced DW motions occur along the nanowire and the magnetic DWs are inverted via the combinations of the spin torques and the IEC. Consequently, the inverted DW can pass from the lower nanowire to the upper PMA nanowire due to its geometry. Due to the strong spin torques, the magnetization along the -*z*-direction is propagated along the +*x*-direction. Because of the strong IEC, the magnetization of the upper layer is automatically inverted to maintain the antiferromagnetic coupling. This is the basic scheme of the SAF-based magnetic inverter. The existence of IEC with the Pt insertion layers deposited at the bottom and top interface of the Ru insertion layer to minimize the contribution of the iDMI energy density should be clearly proven at an early stage of the SAF-type DW inverter development. To verify this issue, magnetic multilayers such as Ta (3.0 nm)/Pt (5.0 nm)/Co (1.0 nm)/Pt (0.2 nm)/Ru (0.9 nm or 1.5 nm)/Pt (0.4 nm)/Co (0.8 nm)/Pt (3.0 nm) are grown by using the DC magnetron sputtering system (see Figure 5b). Polar magneto-optical Kerr effect (MOKE) measurements are performed for the two systems. Synthetic antiferromagnetic (SAF, blue circles) and ferromagnetic (SF, red circles) coupling are observed with two different Ru thicknesses of 0.9 nm and 1.5 nm. We believe that these magnetic multilayer systems with IEC and without the iDMI contribution can provide improved functionalities for the next-generation spin-torque-based logic gates and other related devices.

## 4. Conclusions

In summary, the malfunction mechanism of the magnetic DW-based STMG devices due to the iDMI energy densities is numerically demonstrated. For optimizing the magnetic heterostructure for the next-generation STMG devices, we develop perpendicularly magnetized multilayers with the suppression of the iDMI via a Pt insertion layer. The *D*_s_ is dramatically suppressed by the Pt insertion layer, from 1.23 ± 0.04 to 0.43 ± 0.19 (*t*_Pt_ = 0.2 nm) and 0.48 ± 0.06 (*t*_Pt_ = 0.4 nm) pJ/m. While *K*_s_ is slightly increased, from 0.84 ± 0.01 to 0.97 ± 0.02 (*t*_Pt_ = 0.2 nm) and 0.99 ± 0.03 (*t*_Pt_ = 0.4 nm) mJ/m^2^. Furthermore, we investigate the synthetic antiferromagnetic and ferromagnetic coupling controlled by the Ru thickness in the Pt/Co/Pt multilayer structure. Finally, a different method to demonstrate a magnetic DW inverter by using an SAF structure is proposed.

## Figures and Tables

**Table 1:** Experimentally obtained values of *M*_s_, *K*_s_, and *D*_s._

	Pt = 0.0 nm	Pt = 0.2 nm	Pt = 0.4 nm
*M*_s_ (k/Am)	982 ± 47	1029 ± 28	1049 ± 33
*K*_s_ (m/Jm^2^)	0.84 ± 0.01	0.97 ± 0.02	0.99 ± 0.03
*D*_s_ (p/Jm)	1.23 ± 0.04	0.43 ± 0.19	0.48 ± 0.06

## Data Availability

Data are available upon request.
